# Are Baby Boomers Healthier than Generation X? A Profile of Australia’s Working Generations Using National Health Survey Data

**DOI:** 10.1371/journal.pone.0093087

**Published:** 2014-03-26

**Authors:** Rhiannon Pilkington, Anne W. Taylor, Graeme Hugo, Gary Wittert

**Affiliations:** 1 Population Research and Outcome Studies, Discipline of Medicine, The University of Adelaide, Adelaide, South Australia, Australia; 2 Australian Population and Migration Research Centre, The University of Adelaide, Adelaide, South Australia, Australia; 3 Discipline of Medicine, The University of Adelaide, Adelaide, South Australia, Australia; 4 Freemasons Foundation Centre for Men’s Health, University of Adelaide, Adelaide, South Australia, Australia; CUNY, United States of America

## Abstract

**Background:**

To determine differences in sociodemographic and health related characteristics of Australian Baby Boomers and Generation X at the same relative age.

**Methods:**

The 1989/90 National Health Survey (NHS) for Boomers (1946–1965) and the 2007/08 NHS for Generation Xers (1966–1980) was used to compare the cohorts at the same age of 25–44 years. Generational differences for males and females in education, employment, smoking, physical activity, Body Mass Index (BMI), self-rated health, and diabetes were determined using Z tests. Prevalence estimates and p-values are reported. Logistic regression models examining overweight/obesity (BMI≥25) and diabetes prevalence as the dependent variables, with generation as the independent variable were adjusted for sex, age, education, physical activity, smoking and BMI(diabetes model only). Adjusted odds ratios (OR) and 95% confidence intervals are reported.

**Results:**

At the same age, tertiary educational attainment was higher among Generation X males (27.6% vs. 15.2% p<0.001) and females (30.0% vs. 10.6% p<0.001). Boomer females had a higher rate of unemployment (5.6% vs. 2.5% p<0.001). Boomer males and females had a higher prevalence of “excellent” self-reported health (35.9% vs. 21.8% p<0.001; 36.3% vs. 25.1% p<0.001) and smoking (36.3% vs. 30.4% p<0.001; 28.3% vs. 22.3% p<0.001). Generation X males (18.3% vs. 9.4% p<0.001) and females (12.7% vs. 10.4% p = 0.015) demonstrated a higher prevalence of obesity (BMI>30). There were no differences in physical activity. Modelling indicated that Generation X were more likely than Boomers to be overweight/obese (OR:2.09, 1.77–2.46) and have diabetes (OR:1.79, 1.47–2.18).

**Conclusion:**

Self-rated health has deteriorated while obesity and diabetes prevalence has increased. This may impact workforce participation and health care utilization in the future.

## Introduction

Change in population size and composition, lower workforce participation, demographic ageing, an increase in life expectancy and a rise in chronic conditions are some of the key challenges facing developed countries into the next decades [1]–[6]. The rise in chronic conditions is predicted to impact on workforce participation and health expenditure thereby reducing the tax-base, threatening economic growth and reducing the quality of life of those affected [7]. Baby Boomers comprise 25.3% and Generation X 21.1% of Australia’s population respectively [8]. Given the size of these generations, their continued health into older age is essential to ensure the stability of Australia’s workforce and economy [2]. Baby Boomers, so named following the post-World War II (WWII) rise in fertility were born from 1946 to 1965 (inclusive) [9]. They were aged from 47 to 66 years in 2012 and beginning to enter the retirement phase of life. Those in Generation X were born from 1966 to 1980 (inclusive) and were aged 32 to 46 years in 2012.

For Baby Boomers, the increase in life expectancy since the 1980s has not been matched by improved quality of life, possibly because of the concomitant increase in obesity [7] and associated chronic disease [10]–[13]. In general, Baby Boomers have higher rates of many conditions such as arthritis, osteoporosis, circulatory conditions, overweight, obesity and high blood pressure while Generation X have a higher prevalence of smoking and anxiety, similar levels of psychological distress and better self-rated health [14], [15]. In Australia, as elsewhere, obesity is increasing in younger generations [16] and therefore they may age with a greater burden of chronic disease and poorer quality of life than the generation before them.

The present study examines the health status of Baby Boomers and Generation X at the same age, using 1989/90 and 2007/08 National Health Survey data in order to examine generational differences, irrespective of age.

## Methods

The National Health Survey (NHS) is a population survey designed and conducted in 1989/90, 1995, 2001, 2004/05 and 2007/08 by the Australian Bureau of Statistics (ABS), with the aim of obtaining information on a range of health related indicators. For this study, a comparison of the 1989/90 and the 2007/08 NHS is undertaken.

The NHS was in the field from August 2007 until July 2008. To account for seasonal variation in responses, interviewing times were randomly allocated to four periods. A total of 19,979 households were selected to participate. Following sample loss 17,426 households formed the active sample with a response rate of 90.6% or 15,792 households responding to the survey [17]. A letter and an information brochure, informing the dwelling of the upcoming survey and outlining their right to confidentiality were mailed to all dwellings with complete postal addresses available. Trained interviewers used Computer Assisted Interview technology to collect information about one adult (18 years+) and one child selected randomly from the household [17]. Missing data was not an issue for this analysis.

The 1989/90 NHS was conducted by the ABS from October 1989 to September 1990. A total of 22,200 households were selected at random across Australia. A letter and information brochure was posted to households informing them of their selection for the survey and that an interviewer would be in contact. Trained ABS interviewers interviewed persons aged 18 or older or from 15 to 17 years old with the consent of a parent or guardian, in the selected households. A response rate of 96% was attained [18].

### Variables

Education attainment, employment and smoking status, BMI, physical activity levels, self-rated health and diabetes were able to be matched from the 2007/08 to the 1989/90 NHS, allowing a comparison between the generations. All data are self-report. Education, BMI, smoking and self-rated health were subject to minor recoding to ensure matching categories. Physical activity levels have been calculated by the authors and diabetes was established using differently coded variables. The employment variables did not need to be altered to match.

Education attainment was assessed by asking respondents to provide their current study or highest non-school qualification, if respondents had not completed high school or any qualifications post-high school, they were included in the category ‘no non-school qualification’ [17]. Respondents were classified as employed if they had a job in the week prior to the survey, unemployed if they were actively seeking work and not in the labour force if they met neither of those conditions [17]. Smoking status (tobacco) was categorised into current smokers, ex-smokers if they had smoked at least 100 cigarettes or other at least 20 times and non-smokers if they did not meet this minimum criteria [17].

Physical activity was assessed by asking respondents how much time they had spent walking or doing moderate or vigorous exercise, in the two weeks prior to the survey. The 2007/08 NHS specifically excludes “household chores, gardening or yard work” in their questions on moderate on vigorous activity as types of exercise that could be considered which the 1989/90 survey does not do. However, this was not viewed as a significant barrier to matching the information although it is a potential limitation on comparison. Physical activity levels were defined using the 2008 ABS guidelines [17] and were calculated using the following formula: number of times activity undertaken (in last two weeks)×average time per session (minutes)×intensity. Intensity was defined as 3.5 for walking, 5.0 for moderate exercise and 7.5 for vigorous exercise. Respondents were grouped into four levels according to their score to correspond to sedentary (<100), low (100 to <1600), moderate (1600 to 3200 or >3200 but <2 hours of vigorous activity) and high (>3200 and >2 hours of vigorous activity) levels of physical activity [17].

Self-rated health status was determined by asking respondents if their health is excellent, very good, good, fair or poor [17]. Height and weight were self-report at the time of interview and BMI was defined using Quetelet’s body mass index calculated as weight in kg divided by height (m^2^) [19]. Diabetes status was determined by asking respondents if they had ever been told by a doctor or a nurse that they have diabetes or high sugar levels in their blood or urine [17]. The variable available for the 2007/08 NHS is “Age first told had diabetes or high sugar levels” whilst the variable available from the 1989/90 NHS is “Whether suffers from diabetes or hyperglycaemia”. All respondents who reported an age or indicated they suffered from diabetes or hyperglycemia were classified as having diabetes.

### Analysis

The NHS uses a stratified, multi-staged, area sampling frame of private dwellings and in order to produce unbiased estimates, this sampling technique needs to be taken into account [20]. The sampling unit and stratification information is not included in the datasets released by the ABS, rather a class of techniques called ‘replication methods’ are used to estimate variances for the complex sample design and weighting procedure [17], [21]. The replicate weights are a series of variables that are calculated to account for the design features and their values are based on the sampling and stratification information [22].

Analysis of the NHS data was undertaken using the 2007/08 and 1989/90 Confidentialised Unit Record File [17], [18]. The 2007/08 file contains replicate weights; however the 1989/90 NHS is not released with the replicate weights. In order to ensure these files were comparable the Jackknife (JK-1) method was used to calculate replicate weights for the 1989/90 NHS using STATA IC 11 [22]. JK-1 was the method chosen as this is the method the ABS used for the 2007/08 calculation of replicate weights [17]. The ABS also supplies a person weight, which is adjusted to enable estimation of results for the total Australian population. For example, 20,788 persons were interviewed for the 2007/08 NHS although the data provides weighted population estimates with a total count of 20,643,100.

Applying both the person and replicate weights to the data, cross-tabulations were undertaken to estimate standard errors and proportions. The Z test was used in Microsoft Excel to produce p values adjusted for multiple comparisons using the Sidak method, to compare the variables between the generations for males and females (Table 1).

**Table 1:** A health profile of Generation X (aged 25-44 years) and Baby Boomers (aged 25-44 years) at the same age using 2007/08 NHS data and 1989/90 NHS data from the Australian Bureau of Statistics.

	Males				Females			
	Generation X in 2007/08 (n = 2,949,678)		Baby Boomers in 1989/90 (n = 2,702,515)		Generation X in 2007/08 (n = 2,972,344)		Baby Boomers in 1989/90 (n = 2,658,560)	
	%	S.E.	%	S.E.	%	S.E.	%	S.E.
**Education** #								
Bachelor degree or higher	27.6	0.009	15.2	0.013***	30.0	0.009	10.6	0.009**
Certificate/diploma/trade cert.	37.9	0.010	44.2	0.010***	30.5	0.010	35.3	0.010***
Other/certificate not defined	1.4	0.002	0.8	0.001	1.7	0.002	1.4	0.001
No non-school qualification	33.1	0.010	39.7	0.009***	37.9	0.011	52.6	0.015***
**Employment**								
Employed	89.7	0.007	92.0	0.005*	75.2	0.011	65.7	0.010***
Unemployed	3.0	0.004	4.1	0.003*	2.5	0.003	5.6	0.003***
Not in labour force	7.3	0.006	3.8	0.003***	22.3	0.012	28.7	0.009***
**Smoking**								
Current smoker	30.4	0.009	36.3	0.007***	22.3	0.008	28.3	0.006***
Ex-Smoker	26.0	0.010	23.1	0.005*	25.2	0.009	18.9	0.005***
Never smoked	43.5	0.010	40.6	0.007	52.5	0.009	52.8	0.008
**BMI** #								
Underweight (<18.50)	1.2	0.003	2.0	0.002	2.7	0.003	7.1	0.003***
Normal weight (18.50 to <25.00)	33.2	0.010	52.8	0.008***	45.1	0.011	64.9	0.009***
Overweight (25.00 to <30.00)	37.3	0.010	35.8	0.007	21.8	0.009	17.6	0.006***
Obese (>30.00)	18.3	0.008	9.4	0.005***	12.7	0.006	10.4	0.005***
**Physical activity** #								
Sedentary	32.1	0.010	34.9	0.011	33.2	0.010	35.6	0.011
Low	36.6	0.013	31.6	0.007**	43.0	0.010	39.0	0.008*
Moderate	21.4	0.011	22.6	0.006	19.1	0.009	20.8	0.007
High	9.9	0.008	10.9	0.005	4.7	0.004	4.6	0.003
**Self-rated health (SF36)**								
Excellent	21.8	0.010	35.9	0.009***	25.1	0.010	36.3	0.011***
Very good/good	68.8	0.012	52.4	0.007***	65.8	0.011	51.0	0.009***
Fair	7.6	0.007	10.2	0.004**	7.1	0.007	10.8	0.004***
Poor	1.8	0.003	1.5	0.002	2.0	0.003	1.9	0.002
**Diabetes**								
Yes	2.8	0.003	1.0	0.001**	7.6	0.005	2.9	0.002***
No	97.2	0.003	99.0	0.001**	92.4	0.005	97.1	0.002***

# NA or ‘level not determined’ categories not included.

*p<0.05.

** p<0.01.

***p<0.001.

In 1989/90 Baby Boomers (1989/90 NHS n = 5.3million) were aged 24/25 to 43/44 and in 2007/08 Generation Xers (2007/08 NHS n = 5.9million) were 27/28 to 41/42 years of age. However, due to age only being available in pre-defined groupings, the generations are compared when they were both aged 25 to 44 years.

Logistic regression models were then conducted to adjust for sex, age (5 year groupings), education, smoking status, physical activity and BMI (diabetes model only) when examining the relationship between generation membership, diabetes and overweight/obesity in separate models, from 1989/90 and 2007/08. Table 2 presents results examining overweight/obesity using BMI as the dependant variable and Table 3 presents results examining diabetes as the dependent variable, with generation as the independent variable for both analyses.

**Table 2:** Logistic regression analysis of the association between overweight and obesity (BMI≥25.00) and generation membership of Generation X (aged 25–44 years 2007/08 NHS data) and Baby Boomers (aged 25–44 years 1989/90 NHS data) using data from the Australian Bureau of Statistics.

	Model 1			Model 2 (adj. age, sex, education)			Model 3 (adj. age, sex, education,smoking, physical activity)		
Generation	OR	(95% CI)	p value	OR	(95% CI)	p value	OR	(95% CI)	p value
Baby Boomers	1.00	(ref)		1.00	(ref)		1.00	(ref)	
Generation X	**1.89**	**(1.72–2.09)**	**<0.001**	**2.10**	**(1.82–2.44)**	**<0.001**	**2.09**	**(1.77–2.46)**	**<0.001**

**Table 3:** Logistic regression analysis of the association between diabetes and generation membership of Generation X (aged 25–44 years 2007/08 NHS data) and Baby Boomers (aged 25–44 years 1989/90 NHS data) using data from the Australian Bureau of Statistics.

	Model 1			Model 2 (adj. age, sex, education)			Model 3 (adj. age, sex, education,smoking, physical activity & BMI)		
Generation	OR	(95% CI)	p value	OR	(95% CI)	p value	OR	(95% CI)	p value
Baby Boomers	1.00	(ref)		1.00	(ref)		1.00	(ref)	
Generation X	**1.92**	**(1.60–2.29)**	**<0.001**	**2.05**	**(1.13–3.71)**	**0.019**	**1.79**	**(1.47–2.18)**	**<0.001**

## Results

### Comparisons between Generations of the Same Relative Age using the 1989/90 and 2007/08 National Health Surveys

Education, employment, smoking, BMI, physical activity, self-rated health and diabetes prevalence were examined, by sex, when the generations are at the same relative age of 25–44 years (Table 1), using 1989/90 NHS data for Baby Boomers and 2007/08 NHS data for Generation X.

### Males

Significantly higher proportions of Generation X males reported attaining a Bachelor degree or higher (27.6% vs 15.2% p<0.001), were classified as obese (18.3% vs 9.4% p<0.001), had a low level of physical activity (36.6% vs 31.6% p = 0.002) and reported having diabetes (2.8% vs 1.0% p = 0.001) as compared to Boomer males. As compared to Generation X males, a greater proportion of Baby Boomer males reported being employed (92.0% vs 89.7% p = 0.024), a current smoker (36.3% vs 30.4% p<0.001) and having ‘excellent’ self-rated health (35.9% vs 21.8% p<0.001).

### Females

Generation X females were significantly more likely to have achieved an education level of a Bachelor degree or higher (30.0% vs 10.6% p<0.001), report being employed (75.2% vs 65.7% p<0.001), be classified as overweight (21.8% vs 17.6% p<0.001) or obese (12.7% vs 10.4% p = 0.015) and report having diabetes (7.6% vs 2.9% p<0.001) compared to Boomer females. A higher proportion of Baby Boomer females reported not being in the labour force (28.7% vs 22.3% p<0.001), being a current smoker (28.3% vs 22.3% p<0.001) and having ‘excellent’ self-rated health (36.3% vs 25.1% p<0.001) compared to Generation X females. No differences were demonstrated in physical activity levels.

### Multivariable Analysis

Presented in Table 2, adjusted for sex, education, age, smoking status and physical activity level, Generation Xers had greater odds of being overweight or obese (OR: 2.09, CI95% 1.77–2.46) and presented in Table 3, adjusted for sex, education, age, smoking status, physical activity level and BMI, Generation X had greater odds of diabetes (OR: 1.79, CI95% 1.47–2.18) compared to Baby Boomers, when both generations were aged 25 to 44 years.

When the models were stratified by sex (not shown) the generational difference in diabetes persisted for both males and females in the unadjusted but not in the adjusted analysis. When age and education were included in the model, Generation X females no longer demonstrated greater odds of diabetes (OR: 2.25, CI95% 0.87–5.82) although the difference between Generation X and Boomers males remained significant (OR:1.74, CI95% 1.11–2.74). The generational difference in overweight and obesity remained significant for males and females in unadjusted and adjusted stratified analysis.

## Discussion

Compared at the same relative age of 25 to 44 years Generation X had a higher prevalence of obesity and diabetes compared to Boomers. This was independent of sex, age within that distribution, education, smoking status, physical activity and BMI (diabetes model only). Boomers also demonstrated better self-rated health at the same relative age, although this was unadjusted for demographic factors. This suggests that Generation X may be developing the lifestyle related conditions of obesity and diabetes sooner when compared to Baby Boomers. When the sexes were examined separately, the prevalence of obesity was higher in males as compared to females although the prevalence of diabetes was lower. The difference in obesity prevalence is supported by figures from the Australian Institute of Health and Welfare which demonstrates that males in Australia have a higher prevalence of overweight and obesity compared to females [1]. Despite this, diabetes prevalence was lower in men compared to women, although Australian prevalence data from the ABS illustrates that diabetes prevalence is greater in men [23]. Population studies from England and the USA have demonstrated that prevalence of undiagnosed diabetes is higher in men than in women [24], [25] and a higher prevalence of undiagnosed diabetes among men in this sample may help explain this result although this cannot be confirmed.

When the regression model examining diabetes was stratified by sex and adjusted for age and education, Generation X females no longer had significantly greater odds of diabetes compared to Boomers of the same age. However, despite the non-significance of the result, the odds ratio increased and the confidence intervals widened, suggesting the reduction in sample size and the design effects from the complex sampling strategy the ABS employs, may have been responsible for altering this result for females.

This study adds to the growing evidence suggesting that successive cohorts are developing obesity and related chronic conditions earlier in the life course [26]–[30]. At the same relative age Baby Boomers in the USA [31] and the United Kingdom [32] have been shown to have a greater prevalence of obesity than the older generation (born 1926–1945), associated with more disability and chronic conditions, including diabetes and hypertension. Lee et al. conducted an age, period and birth cohort analysis of individuals in the USA from 1971–2006 and demonstrated that in younger cohorts, obesity is occurring earlier in the life course accompanied by the premature development of conditions such as type II diabetes and arthritis, usually considered to be diseases of ageing [27]. Furthermore, an Australian study examining age, period and cohort contributions to the prevalence of overweight and obesity concluded that more recently born cohorts are at greater risk of overweight [30].

That the younger generation were more likely to report worse self-rated health at the same age as Baby Boomers, may be linked to the significant increase in obesity. Previous studies have demonstrated that obesity, sedentary behaviour and stress are all related to poor self-rated health [33]–[35]. It could be theorised that this is due to comorbid conditions as opposed to weight, although research has demonstrated the association between obesity and self-rated health persists irrespective of chronic condition status [33], [35].

The physical activity and food environment has changed drastically over the past decades to one in which transport options encourage sedentary behaviour and food high in fat and sugar is often more readily available than a healthier alternative [36]–[38]. This may account for why the younger generation are developing an unhealthy weight at an earlier age. Alternative explanations for the cohort differences in obesity include the idea that psychosocial and socioeconomic stressors in early life may play a role in obesity development. The Boomer experience of post WWII prosperity may mean they experienced less psychosocial and socioeconomic stress compared to other generations [28], [39]. Keith et al. also explore the prospect that an increase in sleep debt, endocrine disruptors and maternal age at birthing are plausible contributors to the obesity epidemic [40].

Together, these generations form 76.7% of Australia’s labour force [41] and there is potential for obesity related health-problems to propel an early workforce exit [42]. Should successive cohorts continue to develop what were once considered age related conditions earlier, the consequences for healthcare costs will only increase further, at a younger age [43], [44].

### Limitations

Due to the restrictions in the data granted from the ABS, we were not able to match the generational cohorts by exact birth years for the NHS analysis. Therefore, the ages the cohorts were compared at do not perfectly reflect the true birth years. Although the effect of this on observed generational differences is difficult to estimate, the balance of the age group is made up of the generations in question. We believe that this enables us to make inferences about generational differences although it would have been ideal to examine exact birth cohorts. Additionally, income and alcohol consumption could not be examined for the same age analysis due to significant alterations in the manner the survey assessed the variable. Self-report data was used to calculate BMI and this may have resulted in an underestimation of overweight and obesity, as individuals are prone to underestimate their weight and overestimate their height [45]. All other variables were also derived from self-reported information and this has inherent limitations in terms of potential for social desirability bias and issues with inaccurate recall. Physical activity in particular may be vulnerable to inaccuracies created by individual perception of what constitutes moderate or vigorous exercise [17]. Furthermore, the difference in the physical activity question specification for the 1989/90 and 2007/08 surveys may have affected responses to the questions and therefore this comparison should be interpreted with caution.

Despite this, the generational perspective provides important insights into the development of health in the cohorts across the time span and matches a large range of variables across the NHS surveys.

## Conclusion

Generation X are becoming obese and developing a higher prevalence of diabetes at an earlier age than their predecessors and this may be reflected in their self-reported health status. The current study adds to previous research [26], [27], [30], [46], demonstrating successive generations are developing chronic conditions earlier. If this is to continue there will be significant implications for workforce capacity, health care utilisation and therefore health costs. There is a clear need for continued investment in preventative strategies targeting lifestyle chronic conditions, particularly programs and policies to tackle the increase in unhealthy weight at a population level.
